# The Effectiveness of Prehabilitation (Prehab) in Both Functional and Economic Outcomes Following Spinal Surgery: A Systematic Review

**DOI:** 10.7759/cureus.2675

**Published:** 2018-05-23

**Authors:** Alex Gometz, Diana Maislen, Chelsea Youtz, Erinn Kary, Emma L Gometz, Stanislaw Sobotka, Tanvir F Choudhri

**Affiliations:** 1 Neurosurgery, Mount Sinai School of Medicine, New York, USA; 2 New York University, New York University Medical Center, New York, USA; 3 Columbia University, New York, USA; 4 Neurosurgery, The Icahn School of Medicine at Mount Sinai, New York, USA

**Keywords:** rehabilitation, physical therapy, spinal surgery, back pain, neuropathic low-back pain, preoperative planning, strengthening, prehab, prehabilitation

## Abstract

Rehabilitation prior to orthopedic surgery (prehab) has been studied with more frequency and studies have shown reduced costs and improved functional outcomes among patients who have undergone total hip arthroplasty (THA) and total knee arthroplasty (TKA). This literature review is to determine whether prehab improves functional outcomes and reduces costs following spinal surgery.

PubMed, CINHAL via EBSCO and EMBASE via Ovid were searched with publication date restrictions from May 2006 to May 2016 for the terms ‘physical therapy’, ‘physiotherapy’, ‘prehabilitation’ or ‘prehab’, ‘spine’ or ‘spinal’, and ‘preoperative’ or ‘pre-op’.

The search yielded 737 eligible articles which were screened by two independent reviewers. Randomized controlled trials (RCT) with adults who participated in preoperative exercise interventions as part of a prehab or preoperative exercise program for spinal surgery versus standard care were included.

Methodology and results of the studies were critically appraised in conformity with PRISMA guidelines.

Three RCTs were included, all of which analyzed outcomes of prehab following lumbar spinal surgery. Two of the articles were of high quality and three were of low quality. None of the studies demonstrated a statistically significant difference in pain scores or disability questionnaires in the intervention groups postoperatively, however, no negative effects were reported either. With neuroscience education, patient’s reported feeling prepared for surgery and expressed positive outlook regarding the intervention. Two of the studies found perioperative intervention reduced the total cost of healthcare spending associated with spinal surgery. Due to the heterogeneity of the outcome measures, a meta-analysis was not possible.

There is lack of significant evidence looking at functional outcomes using physical therapy prior to spinal surgery. Prehab should continue to be researched prior to spinal surgery to determine effectiveness in patient outcomes.

## Introduction and background

In 2012, musculoskeletal pain was present in 52.1% of individuals over 18 years old in the United States [1]. Low back pain (LBP) was the most prevalent at 28.6%, followed by knee pain at 18.1%, and neck pain at 15.2%. The number of physician visits involving a complaint of back pain increased from 44.6 million in 2004 to 52.8 million in 2012 [1]. In 2012, the approximate annual direct medical cost for treatment of spine-related problems was $253 billion. This is likely an underestimation due to outpatient treatment, chiropractic care, physical therapy and other alternative care not being included in this approximation [1].

According to Spine-Health, spinal surgery is indicated when a patient’s neck or back pain fails with conservative treatments and becomes disabling [2] but also in the face of progressive neurological deficit or deformity. Spinal decompression (such as a microdiscectomy or laminectomy) and spinal fusion are often the chosen surgical interventions. The purpose of decompression surgery is to relieve pressure on a nerve root by removing the irritating bone or disc material, which has resulted from a herniated disc or spinal stenosis. Spinal fusion, however, involves reducing motion at a painful vertebral segment by insertion or onlay of bone graft, with or without hardware. This procedure is typically indicated for individuals with degenerative disc disease (DDD) or spondylolisthesis [2].

Spinal surgeries especially spinal fusions in the United States increased dramatically in the last two decades from approximately 61,000 in 1993 to over 450,000 in 2011 [3]. The Agency for Healthcare Research and Quality’s Healthcare Cost and Usefulness Project reported an increase in spinal fusions by 40% from 1998 to 2004 [4]. According to the Health Care Utilization Project fusions construct the largest national bill of any hospital-based surgery evaluated at $40 billion [5]. High costs, prolonged hospital stays and surgical readmissions require the construction of improved clinical route and outcomes for the patient [6].

Evidence supporting rehabilitation following spinal surgery is extensive. In a Cochrane Review, Oosterhuis et al. concluded that there is a low-quality evidence that physical therapy after surgery leads to improved function and that multidisciplinary rehabilitation accelerated the subject’s return to work [7]. Short-term pain and functional status were improved with exercise with greater gains when the intervention was high-intensity exercise. None of the studies included in the review reported an increased reoperation rate.

Prehabilitation (prehab) refers to the process of enhancing the functional capacity of an individual in preparation for an anticipated surgical procedure [8]. Theoretically, individuals will be prepared to appropriately handle stresses associated with surgical procedures when they have undergone targeted physical and cognitive training. A generic prehab program includes a warm-up, cardiovascular component, resistance exercises and functional training [9]. Studies have suggested that a physical exercise regimen in the weeks leading up to surgery can improve recovery, physical function, reduce postoperative pain and decrease the length of the hospital stay after orthopedic surgery [10].

A significant link between the benefits of prehab and spine surgery has not been well established as it has been for hip and knee surgeries. Desmeules et al. concluded that prehab was effective in increasing physical function in patients undergoing total hip arthroplasty (THA) or total knee arthroplasty (TKA) by improvement in Lower Extremity Function Score, Self-Paced Walk, Timed Up and Go, and stair test performance following surgery [11]. Calatayud et al. demonstrated that preoperative (pre-op) training improves early post-operative (post-op) outcomes in patients following TKA. Reduced pain and improved strength, the range of motion and functional task performance prior to surgery led to a reduced length of hospital stay and a faster recovery [12]. Brown et al. concluded that patients who underwent prehab exercise prior to a TKA have met their outcome expectations after surgery [13]. Rooks et al. found that a six-week pre-op exercise program prior to THA improved function and strength and reduced the likelihood of discharge to a long-term rehab facility [14].

A systematic review analyzed the effect of a peri-operative physiotherapeutic intervention in individuals with degenerative lumbar conditions awaiting surgery [15]. A few studies suggested a reduction in pain and increased functionality as a response to peri-operative physiotherapeutic intervention. Limitation of high-quality evidence indicates a need for further review of the current literature regarding prehab prior to spine surgeries.

The purpose of this review is to determine whether prehab improves functional outcomes and reduces costs following spinal surgery.

## Review

Methods

A literature search was conducted using the following electronic databases: PubMed, CINHAL via EBSCO and EMBASE via Ovid. The following keywords were used in combination: “physical therapy”, “physiotherapy”, “prehabilitation” or “prehab”, “spine” or “spinal”, and “preoperative” or “pre-op.” A total of 737 studies were identified.

Studies were included for further analysis if they were randomized controlled trials (RCT) where subjects participated in prehab prior to spinal surgery as they produce higher probability. Both lumbar and cervical spine surgeries were included. The underlying disease or disorder that leads to spinal surgery was not specified. Non-English articles were excluded from all searches and only studies examining adult participants were included. Publication dates were limited to the past 10 years, from May 2006 to May 2016.

Screening for study design and relevant abstracts decreased the number of studies included in this review to a total of five articles. Of these five articles, two were studies completed alongside their original RCTs to analyze the cost-effectiveness of the intervention. Therefore, the results of our literature search yielded three distinct experimental protocols but five published articles based on RCTs. All studies included analyzed outcomes of prehab following lumbar spinal surgery.

Results

Data from three RCTs (n = 217) were analyzed. These studies compared the results of a perioperative intervention versus standard care for lumbar surgery candidates (LSC). Rolving et al. investigated cognitive-behavioral therapy (CBT) intervention [16,17]. Louw et al. analyzed neuroscience education (NE) intervention [18], and Nielsen et al. examined prehab [19,20]. In the following tables, Table 1 displays details of the studies and interventions, Table 2 describes Pedro score analyses and Table 3 describes the CBT intervention.

**Table 1:** Description of studies. DDD: Degenerative disc disease; LBPRS: Low back pain rating scale; HRQOL: Health-related quality of life; LS: Lumbar spine surgery; NPRS: Numeric pain rating scale; Q: Questionnaire; ODI: Oswestry disability index; QALY: Quality-adjusted life years; wk: week; CBT: Cognitive behavioral therapy; PT: Physical therapy; D/C: Discharged; POD: Post-op day; NE: Neuroscience education.

	Nielsen, 2008 [20]	Nielsen, 2010 [19]	Rolving, 2014 [17]	Rolving, 2015 [16]	Louw, 2014 [18]
Participants	n = 60 (received intervention) Control: 32 Experimental: 28 Dropouts: 0	Same as Nielsen, 2008 [20]	n = 90 (baseline measures) Control: 31 Experimental: 59 Dropouts: C: 3, E: 4	Same as Rolving, 2014 [17]	n = 67 Control: 35 Experimental: 32 Dropouts: C: 2, E:4
Inclusion criteria	>18 years old	Same as Nielsen, 2008 [20]	DDD or spondylolisthesis grade 1 or 2, 18-64 years old, fusion of max three adjacent vertebrae	Same as Rolving, 2014 [17]	Scheduled for LS for radiculopathy
Exclusion criteria	General contraindications to surgery	Same as Nielsen, 2008 [20]	Surgery scheduled <4 wk after inclusion, >80 km drive to hospital, psychiatric, inflammatory or malignant disease	Same as Rolving, 2014 [17]	Under 18 or older than 65 years, scheduled for LS with instrumentation, participation in pain management program, LS for condition other than radiculopathy, chronic pain condition, symptoms of cord compression
Outcome measures	Pain: Brief Pain Inventory Q Function: Roland Morris Q Sit-to-stand Timed up and go Milestones achieved under hospitalization HRQOL: 15-D	Costs	Pain: Back and leg pain of LBPRS Function: ODI Return to work Psyc: Fear Avoidance Belief Q Catastrophic subscale of Coping Strategies Q Costs: Return to work	Function: QALY ODI Costs	Pain: Leg and back pain by NPRS Function: ODI Psyc: Thoughts and beliefs about surgery Costs: Health care utilization
Control intervention	- Educated about cessation of smoking, harm of drinking, anesthesia, pain management, diet and PT - Mobilized day of surgery and 30 min PT each following day with intention to D/C POD 8 - Pain treatment 12 mg ropivacaine and 6 ug sufentanil per hour		-Pre-op education about the operation, anesthetic procedures, medications, post-op rehab and physical restrictions Post-op rehab including eight weeks of supervised exercise beginning 12 weeks after surgery		Usual care regarding pre-op education controlled by following Spine Surgery Education Questionnaire
Experimental intervention	- 6-8 weeks of prehab – individualized home training 30 min daily focused on cardiovascular conditioning and musculature strength of back and abdomen - Educated about cessation of smoking, harm of drinking, anesthesia, pain management, diet and PT - Smokers received six-week smoking program with free nicotine replacement as well as weekly follow-ups with a nurse - Two weeks before the surgery the patients met with a physical therapist for additional information regarding the operation, postop mobilization and rehabilitation. - Dietary supplement pre- and post-op - Mobilised day of surgery and 30 min 2x/day PT each following day with intention to D/C POD 5 - Pain treatment 8 mg ropivacaine and 4 ug sufentanil per hour and 6 mg ropivacaine and 3 ug sufentanil up to 3x/hr for breakthrough pain		Four pre-op and two post-op CBT sessions in addition to the standard care		- Usual care regarding pre-op education - Pre-op NE program including one 30 min educational session with PT and NE booklet

**Table 2:** Pedro Score analysis.

	Rolving, 2014 [17]	Rolving, 2015 [16]	Nielsen, 2008 [20]	Nielsen, 2010 [19]	Louw, 2014 [18]
Eligibility criteria	X	X	X	X	X
Random allocations	X	X	X	X	X
Concealed allocation			X	X	X
Group similar	X	X	X	X	X
Blind subjects					
Blind therapists					
Blind assessors					
One key outcome from 85% of subjects	X	X			X
All received treatment or “intention to treat”			X	X	X
Between group statistical comparison	X	X		X	X
Both point measure and measure of variability	X	X	X	X	X
Total *score if eligibility criteria excluded	6/11 (5/10*)	6/11 (5/10*)	6/11 (5/10*)	7/11 (6/10*)	8/11 (7/10*)

**Table 3:** Cognitive behavioral therapy. CBT: Cognitive behavioral therapy

	CBT	Prepare for surgery	Homework
Pre-op 1	- Physical and psychological reactions to stressful situations - The link between thoughts, feelings, bodily reactions and behavior	- What to expect from the operation and the post-op course	- Identify and write down thoughts and feelings in relation to painful or stressful situations. Consider and write down alternative and realistic thoughts
Pre-op 2	- Causes and consequences of pain. The fear-avoidance belief model and the importance of physical activity in reducing pain	- Pleasant activity scheduling and activity pacing - Ergonomic: working posture following surgery	- Identify and write down three activities you used to enjoy. Plan and go through with them considering your pain level. How did it affect your mood and pain level?
Pre-op 3	- The link between thoughts, feelings, bodily reactions and behavior - Negative automatic thoughts and their role in the maintenance of a vicious circle - Active and passive coping strategies	---	- Identify and write down your own coping strategies when in pain and distress - Try to use active coping strategies. How did it affect your pain level?
Pre-op 4	- How to cope with pain and distress in relation to family, friends, and work	- The experiences of a previously operated patient. - Legislation and procedures in the authorities when being on sick leave and in relation to return to work	- Say no to three tasks, that you would usually agree to do, despite not being comfortable doing it - Prompt a friend, colleague or family to give you a positive support remark - Give a friend, colleague or family a positive remark and notice the reaction
Post-op 1	- Reflection of how patients have used the acquired cognitive techniques and coping strategies postoperatively - Using pacing techniques to restart daily activities, hobbies, and work	---	- Goal setting for the next three months. - Use pacing techniques to achieve one or more of your goals
Post-op 2	- Reflection of how patients have used the acquired cognitive techniques and coping strategies during the past three months - Discussion of achievements of previously set goals - Setting future goals - Coping with flare-ups - Returning to work – expectations, worries and how to cope with barriers	---	---

Clinical and economic outcomes

Rolving et al. compared the effects of a standard pre- and post-op treatment versus six CBT sessions for LSC [16]. The 3 hour CBT sessions were conducted in small groups organized by an interdisciplinary team and a previously operated patient. The goal of CBT is to identify and challenge a patient’s maladaptive thoughts and modify feelings and behaviors in order to alter their pain experience. Topics included the interaction of cognitive and pain perception, coping strategies, pacing principles, ergonomic directions, return to work and details about the surgical procedure. Refer to Table 3 for outlines of the CBT intervention. Outcome measures were collected at baseline, three months, six months and one-year post-op. Results showed there were no significant differences between groups in back or leg pain, return to work rate or sick leave during a one-year follow-up. There was no significant difference in Oswestry Disability Index (ODI) scores between groups at the one-year follow-up (p = 0.082), but there was a statistically significant difference in ODI scores between groups at three months (p = 0.003) in favor of the CBT group. Psychological outcome measures including the Fear-Avoidance Beliefs Questionnaire physical activity subscale (FABQ-PA) and Coping Strategies Questionnaire-Catastrophizing scale (CSQ-CAT) demonstrated a statistically significant difference between groups at six months (FABQ-PA: p = 0.01, CSQ-CAT: p = 0.04). No significant differences between groups in back pain, leg pain, return to work rate, sick leave, psychological outcomes or ODI scores during one-year follow-up were found.

In an economic evaluation conducted alongside this RCT, the cost-effectiveness of pre-op and post-op CBT were analyzed, which is shown in Table 4 [17]. The primary outcome measure was Quality-Adjusted Life Years (QALY) based on the EuroQol five dimensions questionnaire (EQ-5D) scores. This measure was taken alongside the above-mentioned outcome measures. Costs considered in this analysis included intervention costs, primary health care, secondary health care (data on services used by each patient), medications, productivity loss (missed days of work) and patient costs. At the one year follow-up, there was a significant difference of 0.071 QALY in favor of the CBT group (p = 0.045). No costs other than those associated with the intervention were statistically significant between groups.

**Table 4:** Economic cost, Rolving. USD: United States Dollar; GP: General practitioner; PT: Physical therapy; ER: Emergency room.

June 2015 conversion of Euro to USD: 1.317987		
Mean costs during 1st year	Experimental group	Control group
Intervention costs	$830.33	0
Primary health care GP: Medical specialist: PT:	$345.31 $55.36 $160.79	$326.86 $71.17 $122.57
Secondary health care Admissions: Outpatient visits: ER: Medication:	$25,570.27 $2,307.80 $9.23 $332.13	$24,190.33 $2,398.74 $21.09 $212.20
Production loss Weeks of sick leave:	$38,635.47	$42,021.38
Patient costs Transportation: Production loss:	$152.89 $803.97	0 0
Total costs	$69,183.77	$69,299.76

Louw et al. inquired about the effects of a pain neuroscience education on patients with chronic radicular LBP prior to lumbar surgery [18]. Both control and experimental groups received a standard pre-op education. The goal of NE is to help facilitate patients in conceptualizing their pain as up-regulation of the nervous system rather than dysfunction of the tissue. Measures were taken at baseline, one month, three months, six months, and 12 months post-op. There were no significant differences between groups in the numeric pain rating scale (NPRS) for leg pain, LBP, or ODI scores at any time. The group that received NE scored significantly better for survey questions “I was fully prepared for the surgery” (p = 0.010), “The preoperative education I received prepared me well for the surgery” (p = 0.001) and “The surgery met my expectations” (p = 0.042).

One year after the surgery total mean healthcare expenditure for the experimental group was 45% less than the control group (p = 0.007). Economic costs are listed in Table 5. The usual care group used more radiographs (47 vs 17, p = 0.015) and physical therapy (394 vs 113, p < 0.001) than the intervention group. Refer to Table 5 for economic results.

**Table 5:** Economic cost, Louw. PT: Physical therapy

Total health care utilization at 12 months post-op	Experimental group (n = 28)	Control group (n = 33)
Imaging	$1,158.57	$1,915.76
Diagnostic tests	$19.64	$295.45
MD visits	$790.00	$1,121.82
PT visits	$389.29	$1,212,12
Chiro visits	$108.18	$62.50
Other	$180.15	$258.57
Total costs	$2,678.57	$4,833.48
Total cost per patient	$95.66	$146.47
# of X-rays	17	47
# of PT visits	113	394

Nielsen et al. examined the effectiveness of prehab and early rehabilitation after spinal surgery [19]. Outcome measures were taken at the time of inclusion, hospital admission, hospital discharge, one month, three months and six months post-op. The intervention group’s recovery period was shorter (1-6 days vs 3-13 days, p = 0.001) and they spent fewer days in the hospital (median 5 days vs 7 days, p = 0.007). They also experienced less pain (p = 0.03), and less LBP intensity (p = 0.02) according to the area under the curve analysis. When comparing satisfaction of the treatment and outcome, more patients from the intervention group responded positively (53.6% vs 21.9%, p = 0.02). No differences were noted in radiating pain, timed-up-and-go, sit-to-stand, Brief Pain Inventory Questionnaire and 15D between groups.

Nielsen et al. estimated the costs of prehab and early rehab after lumbar surgery. Direct costs are listed in Table 6. Primary surgical intervention and post-op care costs were identical between groups; indirect costs were related to loss of productivity until return to work [20]. The number of days to return to work was multiplied by the average Danish salary to calculate indirect costs. There was a difference in direct costs between the intervention group and control group. The intervention group lost fewer days of work and indirect costs were lower. In total, the intervention group costs were less than the control group by 15%. The experimental group had higher pre-op costs due to physical therapy evaluation and treatment, smoking intervention and pain treatment. The experimental group had lower post-op hospital costs mainly due to a decreased hospital stay and no secondary surgery. The authors reported that a revision surgery for one patient in the control group that cost $9,198 [13] accounted for 15% of extra costs.

**Table 6:** Economic cost, Nielsen. hr: hour; PT: Physical therapy; pt: patient; d: day.

	Experimental group (n = 28)	Control group (n = 32)
Pre-op:		
Introduction PT 1 hr Physician 0.16 hr Nurse 0.25 hr	28 Euros (PT and physician)	8 Euros (nurse)
PT training (PT 0.5 hr)	27 Euros	
Smoking intervention Nurse 2.8 hr Equipment/meds	15 Euros (Three patients)	0 Euros
Alcohol intervention Nurse 2.8 hr Equipment/meds	0	0
Optimized pain treatment Physician 0.25 hr	9 Euros	0
TOTAL Pre-op:	79 Euros	8 Euros
Post-op hospital:		
PT training	135 Euros (1 hr 5x)	95 Euros (0.5 hr 7x)
Pain treatment	44 E (0.16 hr nurse, 0.16 hr specialist)	29 E (0.16 hr nurse, 0.08 hr specialist)
Hospital stay Bed price: 164 Euro/d	820 Euros (five days)	1,148 Euros (seven days)
Secondary surgery	0	258 E (1 pt: 8,247 Euros)
TOTAL post-op hospital:	999 Euro	1,530 Euros
Post-op primary care:		
General practitioner 14 Euro/contact	22 Euros (total 43 contacts)	27 Euros (total 61 contacts)
Emergency contact 24 Euro/contact	2 Euros (total 3 contacts)	8 Euros (total 10 contacts)
Private PT (45 Euro/hour)	32 Euros (20 hr total)	94 Euro (total 67 hr)
Medical treatment	40 Euros	1 Euros
TOTAL post-op primary:	96 Euros	130 Euros
TOTAL Direct Costs per patient	1,174 Euros	1,668 Euros

Economical outcomes in patients with perioperative intervention look promising when compared to standard care. In one of the studies, there was no difference reported between total economic costs in control and experimental groups (Rolving et al., see Table 6). However, in two other studies, perioperative intervention reduced total cost of treatment (Louw et al., see Table 6 and Nielsen et al., see Table 6). Unfortunately, limited information and different costs measurements provided in published reports from these studies did not allow us to run more formal meta-analysis and produce forest plot to evaluate the global difference in total costs between treatment and control groups by pooling economical outcomes (with standard deviations) from all three eligible studies.

In Rolving et al., although ODI scores were not significantly different at six months and one year, p-values were nearly statistically significant (p = 0.056 at six months, p = 0.082 at one year) [15]. In Louw et al. NE was not effective for improving pain measured by NPRS or improving function measured by ODI at one, three, six or 12 months postoperatively [17]. Even though the experimental group had lower scores for back pain, leg pain, and ODI at all measurement times except for 12 months (back pain and ODI), these differences were not statistically significant. According to Nielsen et al., results showed prehab and early rehab proved to be effective for improving pain intensity according to the VAS as determined by area under the curve [18]. However, no statistical analysis was directly provided regarding LBP and radiating pain median values for the control or intervention groups. No significant differences were noted in radiating pain, timed-up-and-go, sit-to-stand or Brief Pain Inventory Questionnaire.

Discussion

Although the studies examined in this review did not demonstrate significant improvements in all outcome measures, there were no negative effects from any of the interventions reported.

In Rolving et al., the authors claimed that this study was the first to investigate CBT prior to spinal surgery [15]. The strength of this study was both groups received identical therapy except for the addition of CBT in the experimental group, which would isolate the effects of CBT. Also, authors reported a high follow-up rate in both intervention and control groups. The authors stated lack of blinding participants based on the structure of the study as a limitation. Furthermore, the authors had little control over therapy following surgery due to local standard policies. A noted limitation of this review was that the CBT group received supervision from more medical professionals than the control group, which may have influenced results.

Louw et al. suggested the strength in this study was the different educational content focusing on neurobiology along with pain neurophysiology leading to a better surgical experience of the subjects overall [17]. However, the authors mentioned that a lack of educational reinforcement after the surgery might have limited the outcome of the education session that was done prior to the surgery. The language used to explain the nervous system may have been too complex for the general population, which could be a limitation. Furthermore, the patients received an educational booklet to read on their own without a follow-up, which may have reduced the quality of the control intervention. Lastly, physical therapy sessions were not monitored but may or may not have contributed to the overall physical and mental recovery from surgery.

Nielsen et al. claimed theirs was the first study to analyze the effects of prehab and early rehab following spinal surgery [18]. The authors reported the strength of the study was a low 19% drop-out rate. Compliance was also high with the intervention group, noting that the subjects attended more than 80% of the training days. However, the authors reported weaknesses such as prolonged hospital stay duration and delayed discharge time due to complications, traditions, expectations and staff management. The authors also mentioned the disadvantage of a small number of subjects that were not blinded, which could have led to an overestimation of positive results. It was difficult to determine what factors led to improvements in the intervention group since there were various pain medications, prehab exercise programs, durations and frequencies of PT mobilization post-operatively and protein drink supplements. Another weakness of the study is that prehab was a self-reported home exercise program, which was neither controlled nor monitored by a physical therapist. Furthermore, the details of the standard inpatient rehab program applied to the control group were not specified and neither group’s rehab plan was described after discharge from the hospital. This study also demonstrated flaws in regards to statistical reporting. p-values were only reported in the results for certain parameters; not all outcome measures. Additionally, a minimal relevant difference in length of the hospital was determined by the authors as two to three days without explanation.

In the economic evaluation, it is impossible to determine if the secondary surgery complication in the control group was a random occurrence or if the patient was at increased risk as a smoker and did not participate in the study’s smoking cessation program [19].

This review was limited to studies published in English. Brown et al. reported that prehab prior to TKA affects self-efficacy to exercise (SEE) and outcome expectations to exercise (OEE) [13]. Although no significant differences were found between groups for SEE and OEE scores, the intervention group SEE score showed a trending improvement over the time period while the control group SEE scores worsened. Both Brown et al. and Louw et al. shared an underlying psychological link between prehabilitation, motivation to exercise and results of postoperative recovery.

An overview of previous studies concluded that physical therapy incorporating exercise after spinal surgery led to improved function, pain and faster time to return to work in short-term follow-ups [7]. Several studies analyzing the effects of prehab in conjunction with orthopedic surgery have demonstrated potential functional benefits following surgery [9-15]. A study protocol for an RCT has been recently published that examines the effects of a prehab program on patient recovery following spinal stenosis surgery [21].

Pain and function were analyzed in each of these studies, however, direct comparisons could not be done due to insufficient data and different outcome measures used in each study.

## Conclusions

Research regarding prehab and spinal surgery is still lacking. Studies included in this review examined different aspects of prehab and the outcomes following surgery such as pain, function, and costs. In Louw et al., NE was effective in reducing total healthcare expenditure by 45% compared to the control group. Furthermore, the NE group utilized significantly fewer PT visits as well as less than a third of the PT costs that the control group utilized. In Nielsen et al., intervention costs were 15% lower for the experimental group compared to the control group even though intervention costs were higher during the total prehab period. In Rolving et al., however, CBT did not prove to be economically favorable compared to standard treatment.

None of the studies provided definitive evidence supporting prehab based on lack of statistically significant differences in the intervention groups compared to the control groups and lack of standardization of treatment for a fair comparison. It is important to note that none of the participants from the intervention group experienced negative outcomes. Based on this literature review, we can conclude prehab interventions, even though most show preliminary promising results, need to be researched in detail prior to spinal surgery to determine its effectiveness in patient outcomes. Further research is needed to determine if prehab is effective for improving function, pain and reducing cost following spinal surgery. Future studies should incorporate improved methodological format and consistent statistical analysis. These studies should also include a clear description of the prehab intervention so that clinicians can replicate the study if it is shown to be effective.
